# Endovascular treatment of acute ischemic stroke due to anterior circulation large vessel occlusion beyond 6 hours: a real-world study in China

**DOI:** 10.1186/s12883-021-02122-x

**Published:** 2021-02-27

**Authors:** Qing Huang, Mengmeng Gu, Junshan Zhou, Teng Jiang, Hongchao Shi, Xiangliang Chen, Yingdong Zhang

**Affiliations:** 1 Department of Neurology, Nanjing First Hospital, Nanjing Medical University, No.68, Changle Road, Nanjing, Jiangsu Province People’s Republic of China; 2 School of Basic Medicine and Clinical Pharmacy, China Pharmaceutical University, Nanjing, 211198 People’s Republic of China

**Keywords:** Endovascular treatment, Time window, Recanalization, Symptomatic intracranial hemorrhage, Functional outcome

## Abstract

**Background:**

We aimed to assess the safety and efficacy of endovascular treatment (EVT) in patients with anterior circulation emergent large vessel occlusion (ELVO) beyond 6 h from symptom onset in a real-world cohort of patients in China.

**Methods:**

We retrospectively examined 305 patients with anterior circulation ELVO treated with EVT. Patients were divided into two groups: treated with known onset within 6 h (*n* = 238) and beyond 6 h (*n* = 67). Multivariable logistic regression and ordinal shift analyses were used to evaluate the associations between onset-to-groin puncture time and safety and efficacy outcomes.

**Results:**

Treatment beyond 6 h was not associated with symptomatic intracranial hemorrhage within 48 h (sICH; odds ratio [OR] 2.03, 95% confidence interval [CI] 0.48–8.57, *p* = 0.334), in-hospital mortality (OR 1.95, 95% CI 0.48–7.91, *p =* 0.348), successful recanalization (modified Thrombolysis in Cerebral Infarction score 2b or 3; OR 0.73, 95% CI 0.31–1.73, *p =* 0.470), favorable functional outcome (modified Rankin Scale score 0–2; OR 0.55, 95% CI 0.25–1.23, *p =* 0.145), and functional improvement (modified Rankin Scale shift by 1-point decrease; common OR 0.80, 95%CI 0.45–1.42, *p =* 0.450) at 3 months compared with treatment within 6 h. Futher interaction analysis showed that stroke etiology did not modify the associations between onset-to-groin puncture time and outcomes (*p* > 0.05).

**Conclusions:**

In this real-world study, after careful assessment, EVT beyond 6 h from known stroke onset was safe, effective and had comparable short-term outcomes to EVT within 6 h.

## Background

The safety and effectiveness of endovascular treatment (EVT) for acute ischemic stroke (AIS) from anterior circulation emergent large vessel occlusion (ELVO) within 6 h after the onset of symptoms have been fully confirmed by recent large randomized controlled trials [1–5]. The subsequent meta-analysis showed that treatment delay would diminish the beneficial effects of EVT, and the benefit was no longer significant after 7.3 h [6].

More recent trials have shown that patients with AIS beyond 6 h of onset can still benefit from EVT under neuroimaging guidance [7, 8]. The DEFUSE-3 trial [8] (Endovascular Therapy Following Imaging Evaluation for Ischemic Stroke-3) and DAWN trial [7] (Diffusion-Weighted Imaging or Computerized Tomography Perfusion Assessment With Clinical Mismatch in the Triage of Wake Up and Late Presenting Strokes Undergoing Neurointervention With Trevo) extended the time window of EVT to 16–24 h in carefully selected patients. However, the subjects of the two trials were patients with unwitnessed or wake-up stroke, that is, the onset time is actually unknown, and the actual onset time in some cases may still be within 6 h. The real-world experience in western countries demonstrated that EVT beyond 6 h of known symptom onset was feasible and safe and did not increase the risk of symptomatic intracranial hemorrhage (sICH) [9, 10]. Since the main etiology of ischemic stroke in Asian and Western populations are different, in that the Asian population mainly manifested with a higher proportion of large artery atherosclerosis (LAA) stroke [11–13], it is necessary to conduct a real-world study on patients with EVT beyond 6 h in the Asia population.

In this study, we assessed the safety and efficacy of EVT in AIS patients with acute anterior circulation ELVO beyond 6 h after symptom onset in a real-world cohort of patients in China.

## Methods

### Patients

Based on the Nanjing First Hospital Stroke (NFHS) Registry Program [14], we performed a retrospective review of consecutively recruited stroke patients with anterior circulation ELVO (occlusion of the internal carotid artery, and/or middle cerebral artery M1 or M2) treated with EVT between January 2016 and February 2020.

In accordance with the national guidelines, eligible patients were treated with intravenous thrombolysis within 4.5 h after the onset of symptoms. After exclusion of contraindications, patients admitted within 6 h of stroke onset were treated with EVT if they fulfilled the following criteria: 1) 18 years or older; 2) anterior circulation ELVO confirmed by CT angiography (CTA) or MR angiography (MRA); 3) no intracranial hemorrhage or subarachnoid hemorrhage on initial non-contrast CT; and 4) written informed consent. Before 2018, for patients admitted more than 6 h after stroke onset, the further neuroimaging criterion to treat with EVT was an infarction involving less than 1/3 MCA territory with a perfusion-diffusion mismatch ratio ≥ 1.2. And since 2018, the criterion was based on a target mismatch profile defined by the DEFUSE-3 trial (i.e. an ischemic core volume of < 70 mL, a perfusion-diffusion mismatch ratio ≥ 1.8 and a mismatch volume ≥ 15 mL).

According to the purpose of this study, we further excluded patients if they fulfilled the following criteria: 1) with unknown onset of stroke (including unwitnessed and wake-up stroke); 2) had a pre-stroke modified Rankin scale (mRS) score ≥ 2; and 3) had serious heart, liver or kidney dysfunction. Due to the retrospective nature of this study, written informed consent was waived by the ethics committee for the anonymized analysis of data collected as part of routine clinical care in this cohort.

### Clinical characteristics

Data on demographics, stroke risk factors, laboratory indexes, stroke severity, neuroimaging, intravenous thrombolysis, onset-to-groin puncture time, and treatment profiles of EVT were extracted from the NFHS registry database. The etiology of ischemic stroke was classified by the Trial of Org 10,172 in Acute Stroke Treatment (TOAST) criteria [15]. The National Institutes of Health Stroke Scale (NIHSS) score was used to assess stroke severity at admission. Infarct volume after acute anterior circulation ischemic stroke was evaluated by two experienced neurologists (HS and XC) using the Alberta Stroke Programme Early CT Score (ASPECTS) [16, 17]. Discrepancies were resolved by consensus. According to the time from known symptom onset to groin puncture, patients were divided into two groups: within 6 h and beyond 6 h.

### Outcomes

Safety outcomes included in-hospital mortality and sICH confirmed by neuroimaging (CT or MRI) within 48 h. According to the Heidelberg Bleeding Classification, sICH was defined as the newly observed intracranial hemorrhage associated with any of the following conditions: 1) an increase of ≥4 points in total NIHSS; 2) an increase of ≥2 points in one NIHSS category; or 3) deterioration that led to intubation, hemicraniectomy, external ventricular drain placement, or any other major medical/surgical interventions [18].

The recanalization status was classified using the modified Thrombolysis in Cerebral Infarction (mTICI) score [19]. Successful recanalization was defined as mTICI score 2b or 3 and was considered as an efficacy outcome [20].

Short-term functional outcomes were quantified using the mRS score at 90 days, which was evaluated as a routine part of the NFHS registry program through telephone interviews or face-to-face visits. Favorable functional outcome was defined as mRS score 0–2 at 90 days. Functional improvement was defined as mRS shift by 1-point decrease in mRS score at 90 days.

### Statistical analysis

Normally and non-normally distributed continuous variables were reported as mean ± standard deviation (SD) and median [interquartile range (IQR)], and were compared using Student’s *t*-tests and Wilcoxon rank sum test, respectively. Categorical variables were presented as number (proportion) and compared using the Pearson’s *χ*^*2*^ tests.

Multivariable logistic regressions were used to evaluate the associations between onset-to-groin puncture time (≤6 h and > 6 h) and safety outcomes, efficacy outcome and functional outcomes. Multivariable ordinal logistic regression (ordinal shift analysis) was used to investigate the association between onset-to-groin puncture time (≤6 h and > 6 h) and functional improvement (a shift in the distribution of mRS scores) at 90 days. We also investigated whether the etiology of stroke modified the associations between onset-to-groin puncture time and outcomes by adding a multiplicative interaction term in the model.

All regression analyses were conducted with adjustments for the baseline variables reported to have significant effects on safety, efficacy, and functional outcomes, including age, sex, hypertension, diabetes mellitus, atrial fibrillation (AF), smoking, baseline NIHSS, baseline ASPECT score, TOAST classification, occlusion site, fasting blood glucose (FBG), total cholesterol (TC), triglyceride (TG), low-density lipoprotein (LDL) and intravenous thrombolysis.

All statistical analyses were performed using SPSS 23.0 software and a two-sided *p* < 0.05 was considered statistically significant.

## Results

**Fig. 1:**
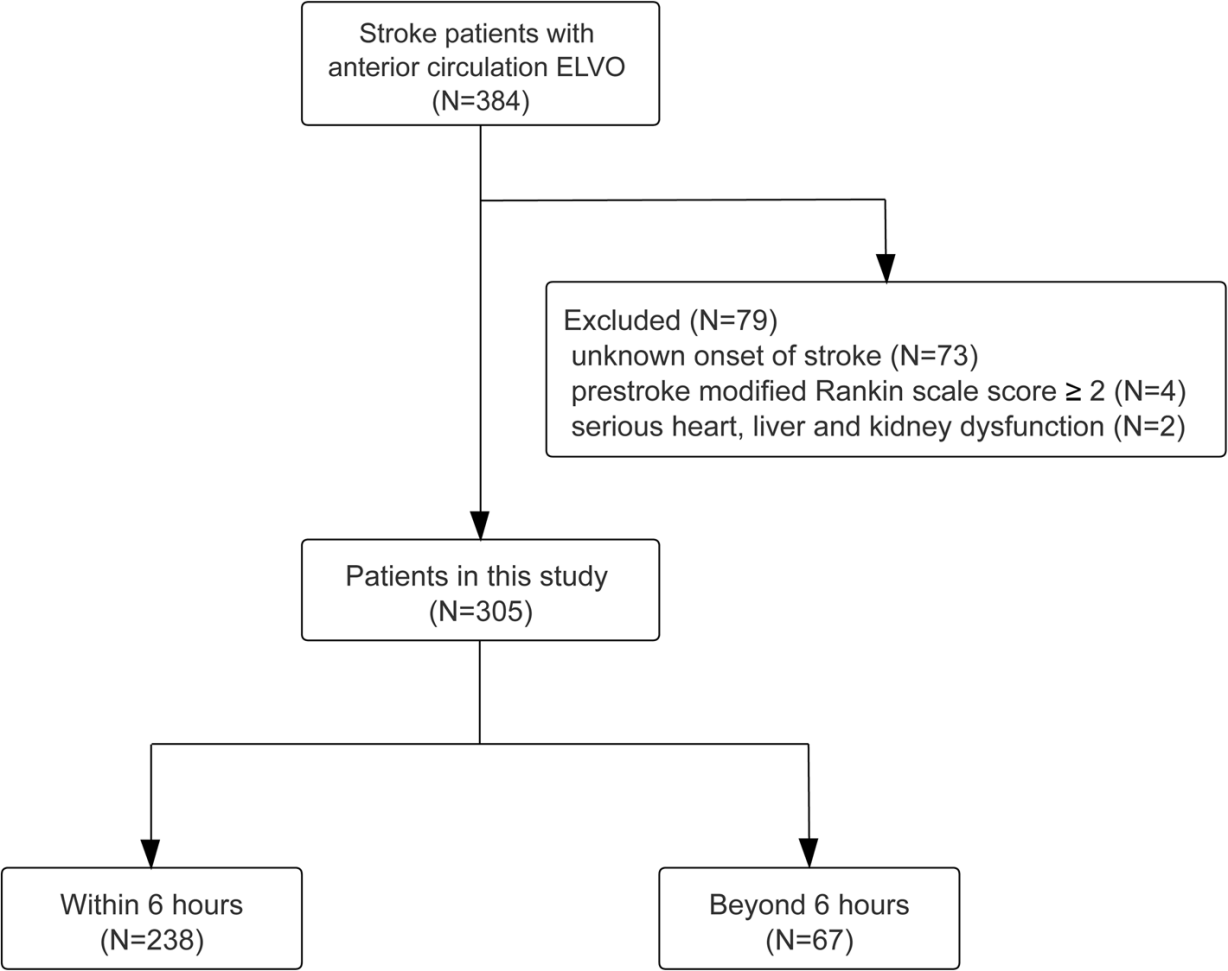
Flow chart for study participants. Abbreviations: ELVO = emergent large vessel occlusion

A total of 384 consecutive stroke patients with anterior circulation ELVO (occlusion of the internal carotid artery, and middle cerebral artery M1 or M2) were treated with EVT between January 2016 and February 2020. Among them, 79 patients were further excluded, and eventually, 305 patients were included in the analysis (enrollment information shown in Fig. 1). Of the 305 enrolled patients, the mean age was 71.1 ± 12.0 years. The median baseline NIHSS score was 16 points (IQR 11–19) and the median ASPECT score was 8 points (IQR 6–9). The median time from stroke onset to groin puncture was 243 (IQR 176–346) min. Successful recanalization was achieved in 257 (84.3%) patients and sICH occurred in 21 (6.9%) patients. The in-hospital mortality was 7.9%. At 90 days, 11 (3.6%) patients were lost to follow-up and favorable functional outcome was achieved in 107 (35.1%) patients.

Of the 305 patients included in the study, 67 (22.0%) were treated beyond 6 h. We compared baseline characteristics of patients treated within 6 h and beyond 6 h (Table 1). Compared with patients treated within 6 h, patients treated beyond 6 h were younger (*p* = 0.001), experienced a less severe stroke (*p* = 0.002), and were less likely to receive intravenous thrombolysis (*p* < 0.001). Vascular risk profiles were also different with a lower incidence of AF (*p* = 0.001), higher TG (*p* = 0.028) and TC levels (*p* = 0.039) in patients treated beyond 6 h. There were also differences in the pathogenesis of stroke (TOAST classification) between the two groups (*p* = 0.006).

**Table 1:** Baseline characteristics of EVT patients treated within and beyond 6 h of onset

	Within 6 h	Beyond 6 h	*p*
	(*n* = 238)	(*n* = 67)	
Age, mean ± SD, y	72.3 ± 1.6	66.9 ± 12.4	0.001
Male, n (%)	135 (56.7)	39 (58.2)	0.828
Hypertension, n (%)	171 (71.8)	46 (68.7)	0.610
Diabetes mellitus, n (%)	53 (22.3)	16 (23.9)	0.781
Atrial fibrillation, n (%)	131 (55.0)	21 (31.3)	0.001
Previous stroke/TIA, n (%)	53 (22.3)	14 (20.9)	0.810
Smoking, n (%)	77 (32.6)	26 (38.8)	0.324
Baseline NIHSS, median (IQR)	16 (12–20)	13 (9–17)	0.002
Baseline ASPECT score, median (IQR)	8 (6–9)	7 (6–9)	0.497
TOAST classification, n (%)			0.006
Large artery atherosclerosis	81 (34.0)	37 (55.2)	
Cardioembolism	134 (56.3)	24 (35.8)	
Others	23 (9.7)	6 (9.0)	
Occlusion site, n (%),			0.746
Internal carotid artery	84 (35.3)	25 (37.3)	
Middle cerebral artery M1	133 (55.9)	38 (56.7)	
Middle cerebral artery M2	21 (8.8)	4 (6.0)	
FBG, mmol/L, (*n* = 299)	6.55 (5.33–8.17)	6.53 (5.55–8.31)	0.516
TC, mmol/L, (*n* = 297)	4.08 (3.37–4.93)	4.35 (3.51–5.12)	0.123
TG, mmol/L, (n = 297)	1.01 (0.74–1.47)	1.20 (0.85–1.68)	0.028
LDL, mmol/L, (n = 297)	2.37 (1.86–3.01)	2.75 (1.92–3.33)	0.039
Intravenous thrombolysis, n (%)	143 (60.1)	11 (16.4)	< 0.001
Onset-to-groin puncture time, median (IQR), min	215.5 (160.0–273.3)	465.0 (395.0–670.0)	< 0.001
sICH	16 (6.7)	5 (7.5)	0.833
In-hospital mortality	18 (7.6)	6 (9.0)	0.709
Successful recanalization	202 (84.9)	55 (82.1)	0.58
Favorable functional outcome at 90 days, (*n* = 294)	85 (37.0)	22 (34.4)	0.704

*Abbreviations*: *SD* Standard deviation, *TIA* Transient ischemic attack, *NIHSS* National Institutes of Health Stroke Scale, *IQR* Interquartile range, *ASPECT score* Alberta Stroke Program Early CT Score, *TOAST* Trial of Org 10,172 in Acute Stroke Treatment, *FBG* Fasting blood glucose, *TC* Total cholesterol, *TG* Triglyceride, *LDL* Low-density lipoprotein, *sICH* Symptomatic intracranial hemorrhage

**Fig. 2:**
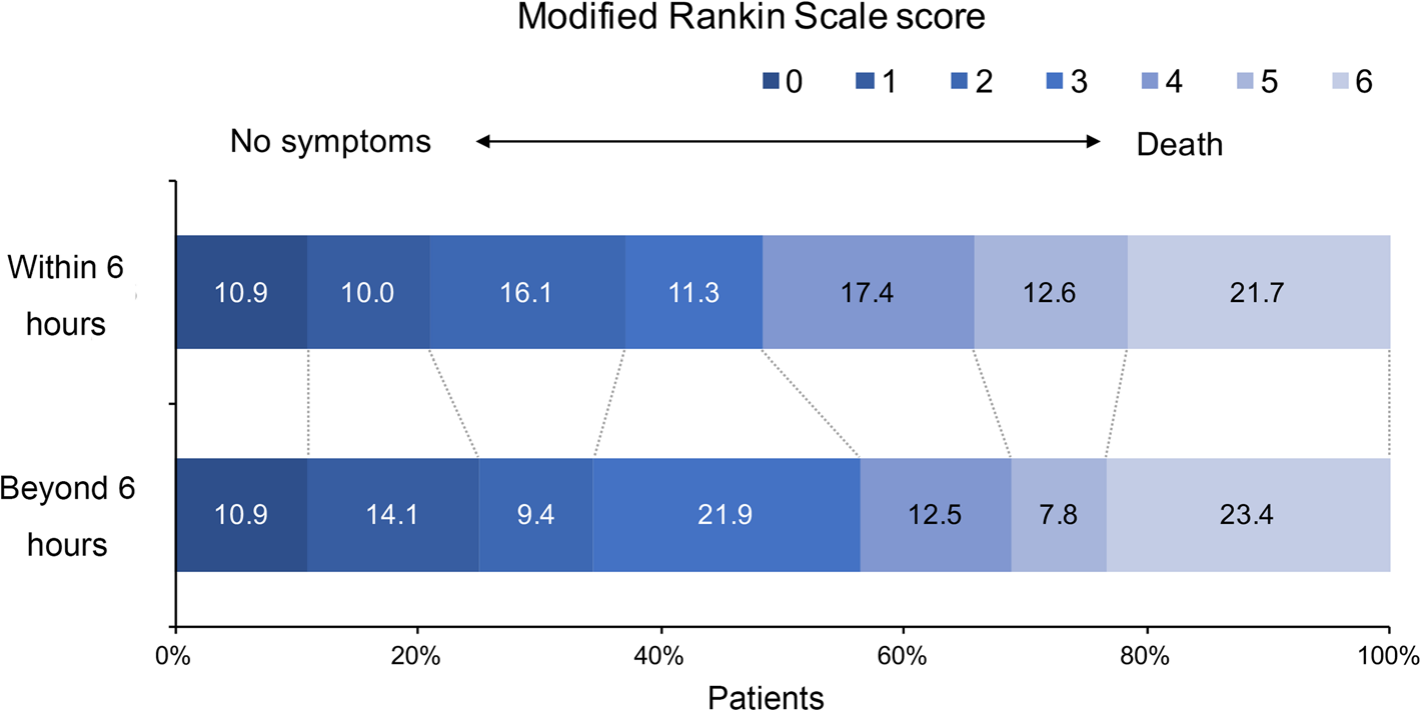
Distribution of 90-day modified Rankin Scale Scores for patients treated with EVT within and beyond 6 h

The comparison of outcomes and the distribution of mRS scores in patients treated within and beyond 6 h were reported in Table 1 and Fig. 2, respectively. No significant differences were found regarding safety (*p* = 0.833 for sICH; *p* = 0.709 for in-hospital mortality), efficacy (*p* = 0.580 for successful recanalization) and short-term functional outcomes (*p* = 0.704 for favorable functional outcome at 90 days) between the two groups.

After adjusting for potential confounding factors, multivariable logistic regression analysis showed that the probability of successful recanalization (odds ratio [OR] 0.73, 95% confidence interval [CI]: 0.31–1.73, *p* = 0.470, Table 2) and 90-day favorable functional outcome (OR 0.55, 95% CI: 0.25–1.23, *p* = 0.145) in patients treated beyond 6 h were lower than those treated within 6 h, but the differences were not statistically significant. There was also no significant increase in the odds of sICH (OR 2.03, 95% CI: 0.48–8.57, *p* = 0.334) and in-hospital mortality (OR 1.95, 95% CI: 0.48–7.91, *p* = 0.348) in patients treated beyond 6 h. The result of ordinal shift analysis showed that the common OR of functional improvement was 0.80 (0.45–1.42), indicating that EVT within 6 h was associated with higher odds of functional improvement, although it was not statistically significant (*p* = 0.450, Table 2).

**Table 2:** The associations between onset-to-groin puncture time (within/beyond 6 h) and outcomes

Outcomes	Unadjusted model		Adjusted model^a^	
	OR (95%CI)	*p*	OR (95%CI)	*p*
sICH	1.12 (0.39–3.18)	0.833	2.03 (0.48–8.57)	0.334
In-hospital mortality	1.20 (0.46–3.16)	0.709	1.95 (0.48–7.91)	0.348
Successful recanalization	0.82 (0.40–1.68)	0.581	0.73 (0.31–1.73)	0.470
Favorable functional outcome at 90 days	0.89 (0.50–1.60)	0.704	0.55 (0.25–1.23)	0.145
Functional improvement at 90 days	1.10 (0.68–1.78)	0.705	0.80 (0.45–1.42)	0.450

*Abbreviations*: *OR* Odds ratio, *CI* Confidence interval, *sICH* Symptomatic intracranial hemorrhage

^a^adjusted for age, sex, hypertension, diabetes mellitus, atrial fibrillation, smoking, baseline NIHSS, baseline ASPECT score, TOAST classification, occlusion site, FBG, TC, TG, LDL and intravenous thrombolysis

The results of interaction showed that TOAST classification did not modify the associations between onset-to-groin puncture time and outcomes (*p* = 0.365 for sICH; *p* = 0.195 for in-hospital mortality; *p* = 0.777 for successful recanalization and *p* = 0.767 for favorable functional outcome).

## Discussion

In this real-world observational study, we compared the safety, efficacy, and functional outcomes between patients with anterior circulation ELVO who received EVT within 6 h and beyond 6 h. The results showed that after careful assessment, EVT treatment beyond 6 h did not increase the risk of sICH and in-hospital mortality, nor did it offset the benefits of EVT for successful recanalization, favorable functional outcome, and functional improvement.

Recently, the results of DAWN [7] and DEFUSE-3 [8] studies showed that patients with stroke onset more than 6 h (6–24 h for DAWN and 6–16 h for DEFUSE-3) could still benefit from imaging-guided EVT, although the 6-h therapeutic window was recommended for EVT in the anterior circulation prior to their publication in 2018 [21]. The application of advanced imaging would give rise to EVT-eligible AIS patients presenting to a comprehensive stroke center, with 1.7% of total patients qualified for the DAWN clinical trial, and an additional 0.6 to 1% who met the DEFUSE-3 trial registration criteria, which may increase the thrombectomy utilization and maximize patient benefits [22].

Patients treated with EVT beyond the 6-h time window in this series had a probability of favorable functional outcomes similar to those receiving EVT within 6 h. However, in the study of Jung et al., the functional outcomes were worse in the beyond 6 h group than the within 6 h group [23]. One possible explanation was that there were more patients with basilar artery occlusion in the > 6 h group than in the < 6 h group, which might account for the worse outcome. Likewise, Casetta et al. reported a lower probability of favorable functional outcomes in patients treated beyond 6 h [9]. While in contrast, an equivalent rate of favorable functional outcomes at 3 months in the early group (0 to 6 h) was also reported when compared with that of the late group (> 6 h) [24], which was consistent with our findings. Similar rates of good outcomes were observed in patients receiving EVT within and beyond 8 h after stroke onset [25]. In another study, with revascularization as a covariate, onset-to-groin puncture time was not an independent predictor of good outcomes, but it (i.e. time window ≤6 h) became independent when revascularization was excluded from the regression model. Their study reinforced that revascularization was a critical intermediate outcome [26]. Moreover, a recent study by Mokin et al. showed that the good clinical outcomes were identical between patients in the 0–6 h group, 6–16 h group, 16–24 h group and > 24 h group [10]. Therefore, consensus has not been reached in the comparability of favorable functional outcome for EVT beyond 6 h vs EVT within 6 h, which needs to be verified by large-scale clinical trials.

In our study, 7.5% (5/67) of the patients treated beyond 6 h developed sICH, which is within the reported prevalence of sICH (3.7–11%) in EVT beyond 6 h [10, 27, 28]. Besides, EVT beyond 6 h did not significantly increase the risk of sICH compared with EVT within 6 h (7.5% vs 6.7%), along with previous findings demonstrating the safety of EVT beyond the 6-h time window [29, 30]. In the study of Jung et al. [23], there was no significant difference in the incidence of sICH between patients treated with intra-arterial therapy beyond 6 h and within 6 h (5.2% vs 3.7%). Accordingly, a recent multicenter, prospective, and observational study also showed that EVT beyond 6 h was safe with no increase in sICH [9]. Instead of EVT time, factors such as cardiogenic stroke, poor collateral circulation and higher neutrophil-to-lymphocyte ratio have been reported to associate with sICH [31, 32], which could be adopted to the risk management of sICH.

In previous studies by Abilleira et al., cardioembolism seemed to be the main cause of stroke, regardless of whether the puncture time was more than 6 h or not (56.3% for less than 6 h and 43.1% for more than 6 h) [26]. In our study, 56.3% of the patients who received EVT within 6 h had a cardioembolic stroke. However, among the patients who received EVT beyond 6 h, the main cause was LAA, with cardioembolism accounting for only 35.8%. Earlier studies showed a contradictory relationship between the etiology of stroke and revascularization after IVT. In one study, cardioembolic stroke was considered to be easier to achieve recanalization compared with other stroke subtypes [33]. However, another study showed the opposite result: patients with extracranial carotid stenosis had higher rates of successful recanalization when treated with EVT compared to those with cardioembolic occlusions [34]. Taking into account the differences in etiology between the two groups, we further conducted an interaction analysis to investigate whether the etiology of stroke modified the associations between onset-to-groin puncture time and outcomes. The results of interaction showed that the correlation between treatment time and successful recanalization, safety and functional outcomes was not affected by the etiology of stroke.

Our study has several limitations. Firstly, this is a single-center hospital-based study, hence selection bias would be unavoidable. Secondly, some patients were lost to follow-up at 3 months. Considering that the rate of patients lost to follow-up was less than 5%, the quality of this study may not be affected. Finally, we did not include medically managed stroke patients with onset beyond 6 h as the control group, so the clinical efficacy of EVT beyond 6 h was not investigated in this study.

## Conclusions

In the real world, imaging-based assessment could extend the time window for EVT to beyond 6 h without increasing the risk of sICH and in-hospital mortality, nor offsetting the benefits of short-term functional improvement.

## Data Availability

All the anonymized data that support the findings of this study are available from the corresponding author upon reasonable request.

## Abbreviations

EVT: Endovascular treatment
AIS: Acute ischemic stroke
ELVO: Emergent large vessel occlusion
DEFUSE-3: Endovascular Therapy Following Imaging Evaluation for Ischemic Stroke-3
DAWN: Diffusion-Weighted Imaging or Computerized Tomography Perfusion Assessment With Clinical Mismatch in the Triage of Wake Up and Late Presenting Strokes Undergoing Neurointervention With Trevo
sICH: Symptomatic intracranial hemorrhage
LAA: Large artery atherosclerosis
NFHS: Nanjing First Hospital Stroke
CTA: CT angiography
MRA: MR angiography
mRS: Modified Rankin scale
TOAST: The Trial of Org 10,172 in Acute Stroke Treatment
NIHSS: The National Institutes of Health Stroke Scale
ASPECTS: The Alberta Stroke Programme Early CT Score
mTICI: The modified Thrombolysis in Cerebral Infarction
SD: Standard deviation
IQR: Interquartile range
AF: Atrial fibrillation
FBG: Fasting blood glucose
TC: Total cholesterol
TG: Triglyceride
LDL: Low-density lipoprotein
OR: Odds ratio
CI: Confidence interval
